# Primary Angle Closure in a 7-Year-Old Child: Evaluation and Findings With Conventional Ultrasound and Ultrasound Biomicroscope

**DOI:** 10.7759/cureus.79280

**Published:** 2025-02-19

**Authors:** Abdulla K Ahmed, Richard Stutzman, William P Madigan, David Belyea

**Affiliations:** 1 Ophthalmology, George Washington University School of Medicine and Health Sciences, Washington, USA; 2 Ophthalmology, Oregon Health and Science University School of Medicine, Portland, USA; 3 Ophthalmology, Childrens National Hospital, Washington, USA

**Keywords:** angle closure, iridotomy, primary angle closure, pupillary block, ultrasound biomicroscope

## Abstract

Primary angle closure (PAC) is rare in pediatric patients, typically associated with persistent hyperplastic vitreous, congenital cataracts, ocular surgery, retinopathy of prematurity, topical or oral medications, and ocular albinism. This case report describes the youngest documented case of unilateral subacute PAC in a seven-year-old female without these underlying conditions. The patient underwent evaluation using A-scan ultrasound and ultrasound biomicroscopy (UBM), revealing a shallow anterior chamber and a short axial length (20.45 ± 0.02 mm in the left eye). The angle closure was caused by a pupillary block associated with anterior displacement of the crystalline lens, unrelated to increased lens thickness or ciliary body abnormalities. A diagnosis was confirmed and laser iridotomy was performed, successfully resolving the condition. Postoperative UBM measurements confirmed the resolution of angle closure. This case highlights the importance of recognizing and promptly treating angle closure in pediatric patients, even in the absence of typical risk factors.

## Introduction

Primary angle closure (PAC) is a condition characterized by the obstruction of aqueous humor outflow due to appositional or synechial closure of the anterior chamber angle, leading to increased intraocular pressure (IOP) and potential optic nerve damage [1]. While more prevalent in adults, PAC is exceedingly rare in pediatric populations, with limited data on its incidence and presentation [2]. This rarity often results in the condition being overlooked in pediatric patients.

In adults, the most common causes of PAC include biometric features such as hyperopia, small corneal diameter, increased lens thickness, and shallow anterior chambers [3,4]. These anatomical predispositions can lead to pupillary block, the primary mechanism of angle closure in this demographic. Conversely, in children, angle closure is more frequently associated with persistent hyperplastic primary vitreous (PHPV), congenital cataracts, ocular surgery (e.g., scleral buckling), retinopathy of prematurity, or exposure to medications like phospholine iodide or brompheniramine-phenylephrine-phenylpropanolamine [5,6].

Diagnosing PAC in children is particularly challenging. Notably, IOP does not necessarily have to be elevated at baseline, as angle closure can occur intermittently or sub-acutely, especially in cases involving relative irido-lenticular pupillary block [7,8]. Furthermore, children are less likely to report symptoms such as pain, blurred vision, or halos around lights, which are commonly associated with acute angle closure in adults [9]. As a result, the condition may remain asymptomatic and undiagnosed until significant anatomical changes or complications arise. These factors underscore the importance of gonioscopy and advanced imaging modalities in the evaluation of pediatric patients with suspicious findings [10].

We present the case of a seven-year-old girl with unilateral intermittent PAC in the absence of structural abnormalities. By evaluating this case, we aim to highlight unique biometric findings, diagnostic methods, and the resolution of angle closure following surgical intervention.

## Case presentation

**Figure 1:**
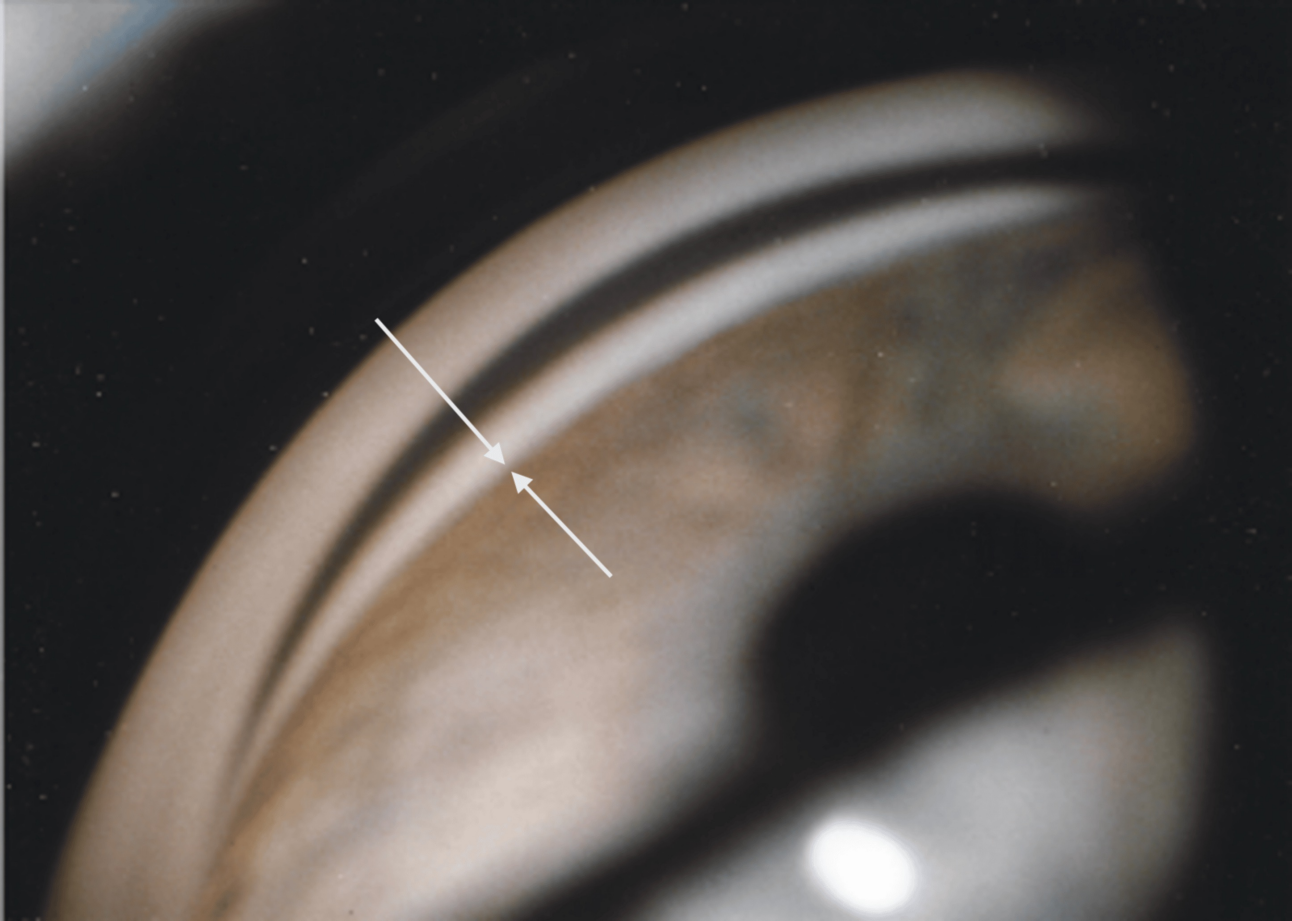
Slit lamp photograph prior to laser iridotomy, showing the iris blocking all angle structures. Gonioscopy, Schaffer grade II.

A seven-year-old Caucasian girl was referred to the eye service due to a history of asymmetric IOP and a previous chalazion in her right eye. Her presenting visual acuity was 20/20 in both eyes (OU) without correction. Goldmann applanation tonometry revealed normal baseline IOP of 14 mmHg OU. Upon slit-lamp examination, the anterior chambers appeared shallow with a convex iris configuration in both eyes. Gonioscopy revealed a Shaffer grade II angle in the right eye (OD) and a narrower angle in the left eye (OS), which was also graded as Shaffer II (Figure 1).

During dilated fundus examination with 2.5% neosynephrine, the IOP increased to 19 mmHg OD and 24 mmHg OS. The cup-to-disc ratio was 0.15 OD and 0.2 OS, with no visible glaucomatous optic neuropathy. Biometric measurements showed a short axial length of 20.45 ± 0.02 mm OS, with normal crystalline lens thickness (3.20 ± 0.02 mm OS) and corneal dimensions (Table 1). Ultrasound biomicroscopy (UBM) of the left eye revealed angle closure due to pupillary block, with no evidence of ciliary body abnormalities, anterior rotation of the ciliary processes, or retro-ciliary effusion.

**Table 1:** Ocular Measurements D = diopters, mm = millimeters, nc = noncycloplegic refraction, OD = right eye, OS = left eye

	OD	OS
Refraction (D)	-0.75+0.75x180^nc^	-0.75+0.75x180^nc^
Mean corneal curvature (D)	45.25	45.37
Horizontal corneal diameter (mm)	11	11
Corneal height (mm)	2.4	2.4
Mean lens thickness (mm)	3.10±0.04	3.20±0.02
Mean axial length (mm)	20.44±0.01	20.45±0.02
Mean anterior chamber depth (mm)	3.00±0.25	2.60±0.03
Relative lens position	0.222	0.205
Lens thickness/axial length factor	1.51	1.66

**Figure 2:**
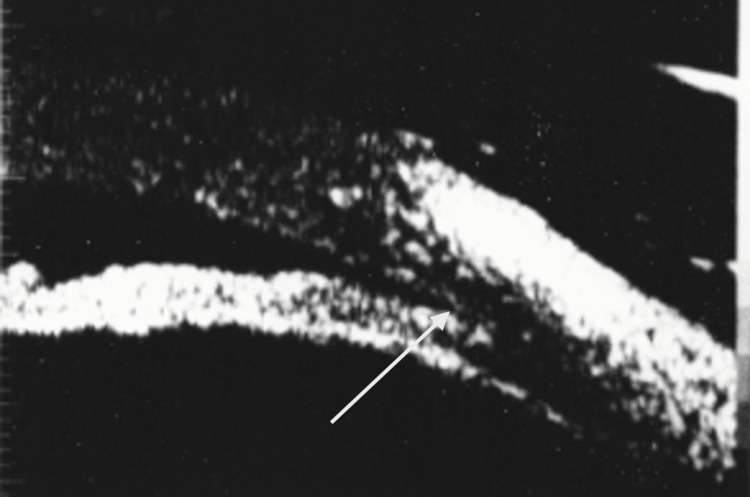
Biomicrograph, OS prior to laser iridotomy, showing angle closure. OS = left eye

**Figure 3:**
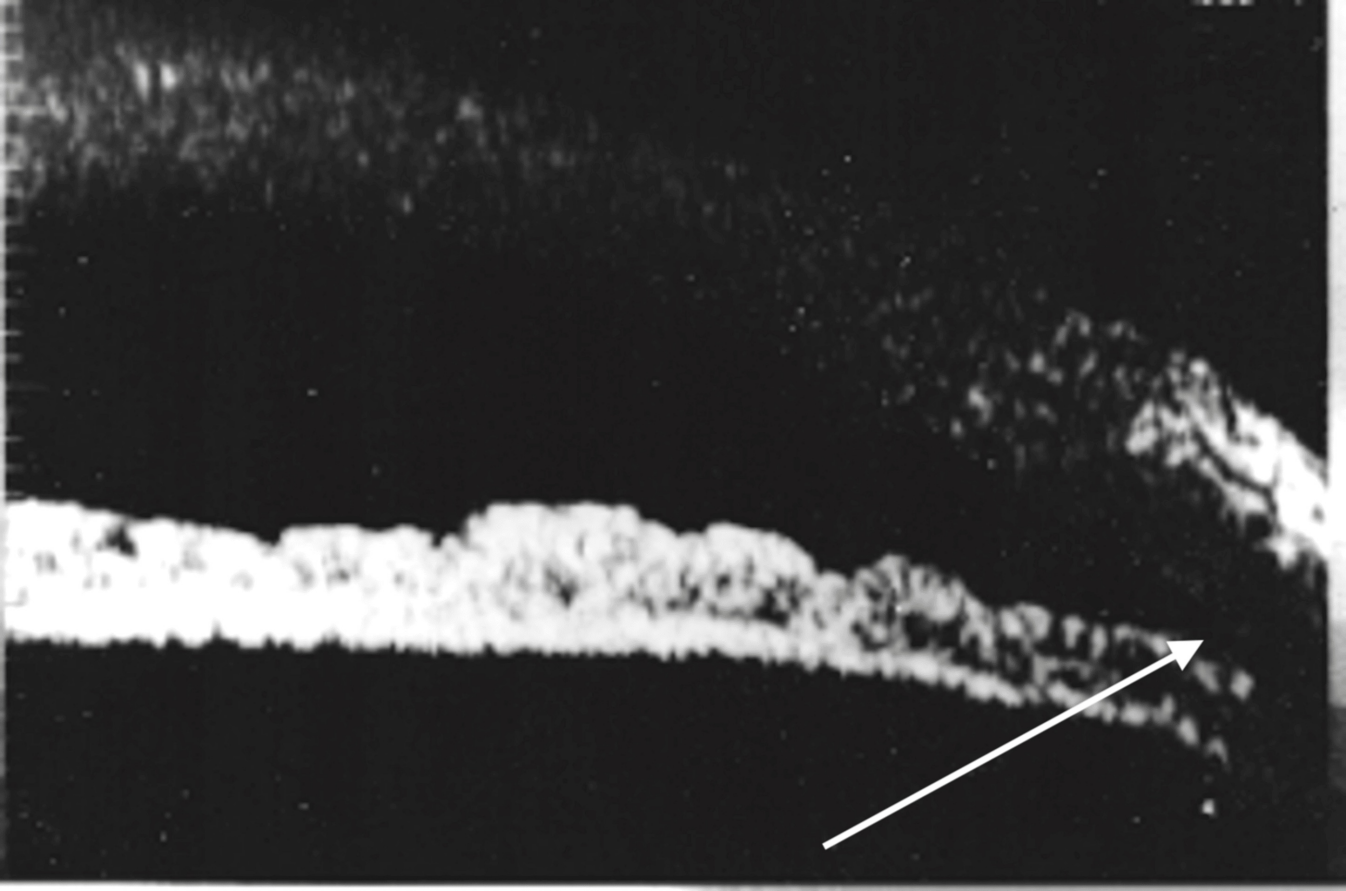
Biomicrograph OS after Nd: YAG laser iridotomy, showing a deeper angle. OS = left eye

A diagnosis of unilateral primary angle closure was made. Laser iridotomy was performed on the left eye (OS), successfully relieving the angle closure (Figure 2, Figure 3). Postoperative gonioscopy demonstrated an open angle extending 360° to the ciliary body band. UBM measurements taken after the procedure confirmed a decrease in anterior lens displacement, with a change in relative lens position (RLP) from 0.205 pre-iridotomy to 0.229 post-iridotomy. Despite recommendations for prophylactic treatment of the right eye, the parents consented to treat only the affected left eye. The right eye remained stable with normal IOP, a narrow angle (Shaffer grade II), and no signs of angle closure during follow-up.

## Discussion

To the best of our knowledge, this represents the youngest reported case of PAC. The seven-year-old patient presented with angle narrowing and IOP elevation due to pupillary block, despite the absence of common predisposing factors. The primary etiology was a short axial length in the left eye (20.45 ± 0.02 mm), which, in combination with anterior displacement of the crystalline lens, led to angle crowding and pupillary block. Importantly, the lens thickness was within normal limits for the patient’s age, and no abnormalities of the ciliary body or other structural anomalies were detected.

The role of axial length in angle closure has been well-documented in adult populations, where shorter axial lengths are strongly associated with an increased risk of angle closure due to anterior segment crowding [11]. Similar to what Tomlinson and Leighton reported, the short axial length in our patient appeared to exacerbate the anterior displacement of the crystalline lens, further reducing anterior chamber depth and increasing the risk of pupillary block [12]. Preoperative measurements revealed a relative lens position (RLP) of 0.205, which improved to 0.229 following laser iridotomy, confirming a significant reduction in anterior lens displacement after the intervention.

Both in normal eyes and those with PAC, shallowing of the anterior chamber angle is known to occur as a dynamic process throughout life, driven primarily by lens growth [13]. This phenomenon can be quantitatively assessed using the lens thickness-to-axial length ratio, also referred to as the lens thickness/axial length factor [14]. Markowitz and Morin reported that this ratio is age-dependent and typically higher in individuals with angle-closure disease than in controls [14]. Interestingly, our patient’s lens thickness/axial length factor (1.66) was lower than the mean values reported in pediatric primary angle closure cases, which range from 1.87 to 2.39 [15]. This suggests that increased lens thickness was not a contributing factor in this case, setting it apart from other documented pediatric and adult cases. Additionally, corneal dimensions, including diameter and height, were within normal limits, further ruling out other potential contributors to angle crowding.

This case highlights the critical role of advanced imaging modalities, such as ultrasound biomicroscopy (UBM), in the evaluation of pediatric patients with suspected angle closure. UBM provided valuable insights into the underlying mechanism of pupillary block, confirming angle narrowing with impaired aqueous outflow prior to surgery and confirming angle opening after laser iridotomy [16]. These findings, supported by gonioscopy, underscore the importance of combining clinical examination with imaging to achieve an accurate diagnosis and guide management. Furthermore, this case emphasizes the need for a high index of suspicion when evaluating pediatric patients with narrow angles or unexplained IOP elevation, even in the absence of systemic or congenital risk factors. The successful resolution of angle closure with laser iridotomy demonstrates the efficacy of this intervention in addressing pupillary block in children. However, the decision not to pursue prophylactic treatment for the unaffected eye highlights the complexities of managing pediatric angle closure, where parental preferences and concerns must be carefully considered.

## Conclusions

In conclusion, this case illustrates that PAC in children can occur due to anatomical variations, such as short axial length and anterior lens displacement, even in the absence of traditional risk factors. Advanced diagnostic tools like UBM are essential for identifying the underlying mechanism and optimizing treatment strategies. Further research is needed to deepen our understanding of the pathophysiology of angle closure in pediatric patients and to develop tailored, age-specific management protocols.
